# Targeted lipid nanoparticles containing IL-10 mRNA improve outcomes in experimental intracerebral hemorrhage

**DOI:** 10.21203/rs.3.rs-6347773/v1

**Published:** 2025-04-08

**Authors:** Sahily Reyes-Esteves, Aparajeeta Majumder, Nicolas Marzolini, Marco Zamora, Yufei Wang, Carolann Espy, Tyler Ellis Papp, Awurama Akyianu, Jia Nong, Lawson Messe, Serena Omo-Lamai, Hamideh Parhiz, Jacob Myerson, Oscar Marcos-Contreras, Jacob Brenner

**Keywords:** intracerebral hemorrhage, endothelial inflammation, targeted nanoparticles, brain drug delivery, hemorrhagic stroke

## Abstract

Intracerebral hemorrhage (ICH) is a lethal and highly morbid form of stroke for which there is no disease-specific therapy. Inflammation after ICH is an important mechanism of secondary damage, and the inflamed endothelium in ICH is a promising therapeutic target as it is the gateway for inflammation in the brain. Systemic therapies that target inflammation have been unsuccessful in stroke, in part due to side effects or poor brain delivery. We hypothesized that targeting mRNA encoding IL-10, a potent anti-inflammatory cytokine, to the brain vasculature would improve outcomes in an experimental mouse model of ICH. We manufactured lipid nanoparticles (LNPs) using microfluidics, packaged them with IL-10 mRNA, and decorated them with antibodies against vascular cellular adhesion molecule (VCAM), which can bind the inflamed brain endothelium after ICH. VCAM LNPs distributed to the brain ~4x more than nonspecific LNPs and expressed their cargo in the brain at 10x higher levels. Treatment with VCAM-LNPs containing IL-10 mRNA led to ~69% reduction in hematoma size at 72 hours after ICH and ~65% improvement in motor behavior in our model, with no improvement in vascular leakage. Finally, we observed that VCAM-LNPs primarily target infiltrated macrophages and neutrophils. VCAM-IL10-LNPs significantly increased plasma and brain levels of IL10. Our data provide preliminary data for a promising therapeutic and delivery strategy for ICH, and shed light on the relationship between inflammation and vascular leakage. Future experiments will seek to understand how serial dosing affects LNP expression in our model and whether treatment at later time points after ICH can still confer therapeutic effects.

## Introduction

Intracerebral hemorrhages (ICH) cause the highest disability and mortality out of all stroke types, and treatment is mainly supportive^1–3^. ICH patients often present to care 2–4 hours after onset, and the hematoma can continue to grow in the first 24 hours^4–7^. Aside from mass effect, secondary mechanisms of injury like inflammation are thought to play a major role in the detrimental consequences of ICH^8,9^. After disruption of the blood brain barrier (BBB) caused by bleed onset, activated endothelial cells recruit peripheral immune cells that contribute to this inflammation^10,11^. This recruitment requires adhesion molecules such as vascular cell adhesion molecule (VCAM), expressed in endothelial cells and leukocytes^12–14^.

Among candidate therapies for immunomodulation after ICH, IL-10 stands out. This potent anti-inflammatory cytokine limits endothelial-leukocyte interactions, decreases oxidative stress, and improves neuronal and glial survival^15–19^. Clinically, higher IL-10 levels in ICH patients correlate with better outcomes at 90 days^20,21^, and preclinical augmentation of IL-10 via recombinant protein delivery also improved behavioral outcomes^22^. However, recombinant IL-10 is quickly cleared from the bloodstream, and systemic delivery in inflammatory disease models has led to off-target effects that have precluded clinical translation^23,24^.

Our prior work showed that despite the vascular leakage seen in ICH, brain accumulation of therapeutic proteins is low when these are delivered systemically via IV injection^25^. However, we were able to greatly improve therapeutic protein delivery to the brain by coupling the cargo proteins to nanoparticles that were conjugated to antibodies against VCAM. This proof-of-concept work showed that VCAM-nanoparticle targeting could greatly exceed passive delivery of therapeutics in experimental ICH.

To build on this, here we use VCAM targeting to deliver mRNA-loaded lipid nanoparticles (LNPs). Delivering mRNA has several advantages over delivering protein: mRNA can produce far greater amounts of protein at the site of targeting; cargo proteins are often damaged when coupled to nanoparticles; and loading multiple different mRNAs is no harder than loading one mRNA, whereas each new protein therapeutic requires unique manufacturing and conjugation to the nanoparticles. As our prototype cargo mRNA, we chose IL-10 mRNA, and a formulation of LNPs that we previously observed is non-inflammatory^26^.

We first show that our VCAM-LNPs express in the brain at 10x the levels of non-specific LNPs. Using our screening strategy for therapeutic efficacy in ICH, we show that our VCAM-LNPs, when loaded with IL-10 mRNA, lead to smaller hematoma sizes and improved behavioral outcomes in the mice. Finally, we show that these LNPs are, surprisingly, not just expressed in brain endothelial cells, but also are highly expressed in infiltrating leukocyte subpopulations. This work marks one of the first publications of targeted mRNA-LNP technology as an efficacious delivery strategy with therapeutic effects in experimental ICH, a devastating human disease with limited therapeutic options.

## Methods

### Reagents.

Lipid reagents for LNPs were purchased from Avanti Polar Lipids (Alabaster, AL). NHS-PEG4-Dibenzocyclooctyne (DBCO) was purchased from Click Chemistry Tools (Scottsdale, AZ). Rat IgG was purchased from Invitrogen. Anti-mouse-VCAM-1 (clone MK2.7) was produced by culturing hybridoma cells, purified via protein G sepharose column (GE Healthcare Bio-Sciences, Pittsburgh, PA) and dialyzed in PBS. For IHC, Anti-CD31 (MEC 13.3) was purchased from Biolegend, Anti-CD45 was purchased from Abcam (ab10558). For flow cytometry, the following antibodies were used: CD31-APC (Thermo Fischer), Ly6G-AF700 (Biolegend), CD64-PECy7 (Biolegend), CD45-BUV395 (BD), CD11b-APCFire810 (Biolegend). mCherry mRNA was commercially purchased from Trilink (San Diego, CA).

### Modification of proteins.

For biodistribution, IgG was directly labeled with [^125^I]Na (Perkin Elmer, Waltham, MA) and Pierce Iodogen radiolabeling reagent. They were purified with Zeba desalting spin columns (Thermo Fisher). We ensured > 90% radiochemical purity via TLC (75% methanol, 25% ammonium acetate). In vivo immunoreactivity was confirmed prior to use via comparison to historical controls. For LNP decoration, VCAM and IgG antibodies were conjugated to DBCO-PEG4-NHS ester by diluting to 15mM and mixing at 1:7 ratio. After 1 hour at room temperature, antibodies were purified using Amincon Ultracel-50kDa membrane filters (Millipore).

### mRNA Synthesis.

DNA sequence for encoding mCherry fluorescent protein was sourced from SnapGene (www.snapgene.com/resources) and then codon optimized. The Murine IL-10 gene sequence was sourced from NCBI (NCBI Reference Sequence: NP_034678.1) and then codon optimized. IL-10 gene sequences were then cloned into an *in vitro* transcribed mRNA (IVT-mRNA) production template plasmid carrying a T7 promoter, 5′ and 3′ UTR elements, Kozak consensus sequence, and 101 poly(A) tail. Synthesis, cloning, and plasmid preparation service was provided by GenScript. IVT-mRNA (*in vitro* transcribed mRNA) was synthesized on linearized plasmids using the T7 MEGAScript Kit (Thermo Fisher) with N1-Methylpseudouridine-5’-Triphosphate (TriLink, N-1081) incorporated into the reaction. Co-transcriptional 5′ Capping of the IVT-mRNA was performed in the reaction using the cap1 analog, CleanCap^®^ Reagent AG (3′ OMe) (TriLink, N-7413). Single-stranded IVT-mRNA was purified by cellulose purification, as previously described (Baiersdörfer, 2019).The mRNA was analyzed by agarose gel electrophoresis and was stored at − 20°C.

### LNP Formulation.

LNPs were prepared using the microfluidic mixing method using the NanoAssemblr Ignite microfluidic device (Precision Nanosystems). LNPs were designed with azide functionalized phospholipids to allow antibody conjugation. An organic phase containing a mixture of lipids dissolved in ethanol at a specified molar ratio was combined with an aqueous phase (50 mM citrate buffer, pH 4) containing nucleoside-modified mCherry and IL-10 mRNA as described above. The mixing occurred at a flow rate ratio of 1:3, with a lipid to mRNA weight ratio of 40:1 and a flow rate of 6ml/min. The resulting LNPs were dialyzed in a 10 kDa molecular weight cutoff cassette in 500 ml of 1× PBS for 2 hours and stored at 4°C. IL-10 mRNA was synthesized as above, and mCherry mRNA was purchased from TriLink Biotechnologies. Mice were dosed based on the concentration of encapsulated mRNA. After concentrating the antibody-bound LNPs, encapsulation efficiency and mRNA concentration were requantified using Invitrogen’s Quant-iT RiboGreen RNA assay. The required volume was determined based on the encapsulated mRNA concentration. Sterilized 1x PBS was used to top off the injection volume to ensure a consistent 100 μl. The dose was administered intravenously via retro-orbital injection.

### LNP characterization.

Following formulation, LNPs underwent stringent quality control to ensure standardization. Hydrodynamic size, particle distribution, and polydispersity index (PDI) were measured via dynamic light scattering (DLS) using a Malvern Panalytical Zetasizer Pro ZS, with any particles exceeding a PDI value of 0.2 excluded. Encapsulation efficiency and concentration of encapsulated mRNA in the LNPs were quantified using Invitrogen’s Quant-iT RiboGreen RNA assay. Particle concentration was determined through nanoparticle tracking analysis (NTA).

### Antibody conjugations.

For conjugation of targeting antibodies (both VCAM and IgG), LNPs were prepared and incubated overnight with either antibodies at 4°C. Immuno-NPs (~ 70mAbs/LNP) were purified using a 20mL Sepharose 4B-Cl column (GE). Antibodies were modified by adding a 5x–7x molar excess of DBCO and a 0.1x molar excess of AF 594, then incubated at room temperature with rotation for 30 minutes. LNPs were fluorescently tagged with AF 594 to assess conjugation efficiency to LNPs.

DBCO modification enabled the antibodies to conjugate to the LNPs via DBCO-Azide copper-free click chemistry. Antibodies were then washed and purified using 10 kDa Amicon centrifugal filter units, centrifuged at 3200 x g for 30 minutes with 1x PBS at 10x the antibody volume. Modified antibody concentration was determined by nanodrop and corrected for DBCO. Based on the concentration of antibodies and concentration of LNPs obtained via NTA, antibodies were added to LNPs and incubated at 4°C overnight to achieve approximately 70 antibodies per LNP. The next morning, a size exclusion chromatography was performed using a Sepharose column to separate LNPs bound antibodies from free fluorophore and unbound antibodies by adding 24 ml of 1x PBS 1 ml at a time. Conjugation efficiency was determined by collecting the different fractions from the column and running it on a fluorescent plate reader. Any background fluorescence was subtracted before calculating conjugation efficiency as a percentage of LNP bound antibody / total antibody. The fractions that contain the LNP bound antibodies were concentrated using 100KDa Amicon filter units by centrifuging at 3200 x g for approximately 25–40 minutes.

### Animals.

Experiments were done according to the NIH Guide for the Care and Use of Laboratory Animals and protocols were approved and overseen by the University of Pennsylvania’s Institutional Animal Care and Use Committee. We used male C57BL/6 mice aged 10–12 weeks and weighing 20–30g (Jackson Laboratory) for all experiments.

### Statistical Analysis.

Analysis was performed using GraphPad Prism 9. Unless specified, two-way ANOVA with post-hoc Tukey’s test was employed.

### Experimental intracranial hemorrhage (ICH) in mice.

Experimental ICH was induced using bacterial collagenase as described^40–42^. Briefly, mice were anesthetized using 2% isoflurane at a rate of 2L/min in 100% oxygen. They were placed in a stereotaxic frame and the skull was exposed. A 1mm hole was drilled at 3mm lateral, 0.5mm anterior from bregma. A 33G needle (WPI) was slowly lowered 3mm ventrally through the opening. After 5 mins, 0.1 units of collagenase I (Gibco) in 600nL sterile saline were infused at a rate of 100nL/min using a syringe pump. After injection, the needle was left in place for an additional 10 mins before slow withdrawal and wound closure.

### Grid walk.

Analysis of motor deficit in mice after acute ICH was performed using grid walk (or foot-fault) testing, as described^43,44^. Briefly, animals were placed on an elevated grid with 1.69cm^2^ square openings and allowed to freely move across the grid. Their movements were recorded for 3 mins via a digital camera for subsequent analysis. Percent ratios of foot-fault over total number of steps for the ipsilateral versus contralateral hind paws were calculated. Statistical analysis was performed using a two-way ANOVA with a post-hoc Sidak’s multiple comparisons test.

### Hematoma quantification.

Intracerebral hematomas were quantified as previously described^8,45,46^. Briefly, after perfusion with 1X PBS, brains were removed from the skull and fixed in 10% formalin. 1mm coronal sections were prepared using a mouse brain matrix (Braintree) and layered onto a slide. Images were then captured using a digital camera and quantification of hematoma volume in each section was done using FIJI (NIH), from which volume was calculated by the sum of all slice volumes.

### Fluorescent microscopy.

After experimental ICH, Ai6 mice (Jackson Labs) were injected with VCAM-LNPs containing cre-recombinase mRNA daily for 3 days. After sacrifice and perfusion with PBS, brains were freshly frozen for 10 um thick cryosections. Tissue was then fixed with 4% paraformaldehyde and imaged using a Zeiss LSM 980 confocal microscopy.

### Radiotracing and biodistribution of antibodies and drug candidates.

For radiotracing biodistribution experiments, mice were injected with 5ug mRNA (or 2 million cpms for radioactive dose) at various times after ICH, as described in the results. After circulating for 30 mins, animals were anesthetized and perfused prior to organ harvesting. Radiotracing was done using a Wizard 2470 gamma counter.

### Vascular Leakage.

To measure vascular leakage, injured animals were injected at various time points after collagenase injection with ^125^I-radiolabeled bovine serum albumin. For all time points, albumin was allowed to circulate for 4 hours prior to sacrifice. Brain vascular leakage was calculated after perfusion by examining the ratio of radioactivity per gram of brain over radioactivity per gram of blood.

### Brain flow cytometry.

Brains were dissociated as described^47,48^. Briefly, brains were first manually disaggregated using repeated pushing of tissue through 18G and subsequently, 21G needles. The resulting suspension was filtered through a 100 μm nylon strainer, centrifuged, and resuspended in dispase (2.5 U/mL, ThermoFisher) for 1 hour. Afterwards, the suspension was passed through a 70 μm filter, treated with 600 units/mL of DNase I (grade I, Sigma Aldrich) prior to centrifugation and demyelination of cell pellet using a standard isotonic Percoll (SIP) gradient. Afterwards, the resulting pellet was suspended in the ACK lysis buffer (Quality Biological) for RBC lysis. Cells were stained with fluorescent antibodies. Flow cytometry was performed using a BD LSR Fortessa Cell Analyzer. Data was analyzed via FlowJo.

### Luminescence.

LNPs containing luciferase mRNA were fabricated as above, injected IV into mice and allowed to circulate for 4 hours. Afterwards, animals were perfused with PBS and organs were flash-frozen prior to homogenization. On the day of analysis, samples were loaded into 2 mL microtubes and suspended in 900 μL of the homogenization buffer (5 mM EDTA, 10 mM Tris, 1:100 diluted protease inhibitor (Sigma), 1X PBS). A single stainless steel 5mm bead (Qiagen) was then added to each sample, and all microtubes containing samples were then placed in a tissue homogenizer (Powerlyzer 24, Qiagen) (2000 RPMs, 2 cycles, 45 s per cycle, 30 s pause in between cycles). After homogenization, 100 μL of lysis buffer (Promega) was added to each tube followed by rotation at 4°C for one hour. Samples were next quickly transferred into new tubes and sonicated (amplitude power 30%, 5 cycles of 3 sec on/off for total of 15 s sonication per sample) to break down DNA and prevent excess viscosity. Once sonicated, samples were centrifuged for 10 min (16,000 G, 4°C). Supernatant was then collected and transferred into new tubes. To quantify luminescence, 20 μL of tissue lysate homogenate was transferred into a 96-well plate, and 100 μL of luciferin solution (Promega) was added to each sample well immediately prior to reading in the luminometer (Wallac). Finally, a Lowry assay (Bio-Rad) was performed using diluted samples (1:40 for all organs except liver, which was diluted 1:80 in 1X PBS). Final luminescence values were normalized by total protein concentration obtained from this Lowry assay.

### In vivo cytokine measurements.

Cytokine measurements were carried out in plasma collected from ICH mice using the LegendPlex 13-plex Mouse Inflammation Panel (Biolegend) per manufacturer’s protocol. Cytokine measurements were also carried out in brain tissue collected from ICH mice using ELISA IL-10 kit (Abcam) per manufacturer’s protocol.

## Results

### VCAM-targeted LNPs have superior brain delivery and expression in ICH-injured mice when compared to non-specific control.

**Figure 1:**
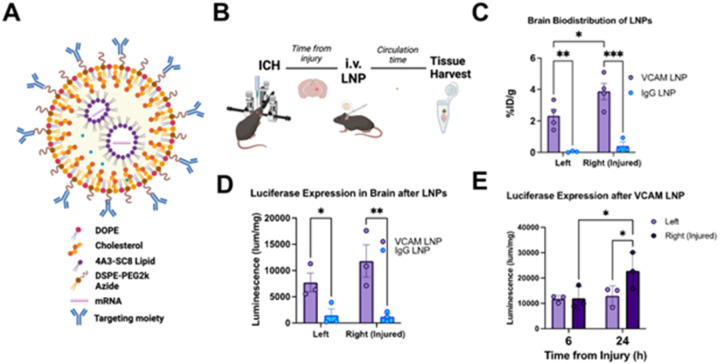
In ICH mice, VCAM-targeted LNPs have better brain distribution and mRNA expression than non-specific control. (A) Graphical schematic showing our LNP formulation containing 4A3-SC8 (ionizable lipid), Dioleoylphosphatidylethanolamine (DOPE, helper lipid), 1,2-distearoyl-sn-glycero-3-phosphoethanolamine (DSPE)-PEG2k azide, and cholesterol. (B) Procedure for experimental ICH, biodistribution, and expression of VCAM-targeted LNPs. ICH was induced via stereotactic injection of collagenase into the brain’s striatum. After injury, VCAM-targeted and IgG LNPs were injected intravenously and allowed to circulate prior to organ harvest. (C) Radiotracing analysis was performed 4 hours post injury with 30 minutes of circulation time and demonstrated significantly higher brain delivery of ^125^I-radiolabeled VCAM-targeted LNPs compared to IgG controls in both right (ipsilateral to injury) and left (contralateral to injury) brain. (D) Measurement of luciferase expression in a separate cohort (treated 2h post-injury with subsequent 4h circulation time) showed that VCAM-targeted LNPs exhibit 6–8x higher expression compared IgG controls. (E) After LNP injection at 2 hours post-injury, time-course analysis of luciferase expression revealed a significant increase at 24h post-injury in ipsilateral hemisphere compared to both contralateral hemisphere at 24h as well as ipsilateral at 4h. For all figures, post-hoc uncorrected Fisher’s least significant difference test was performed after two-way ANOVA. All data represent mean ± SEM, *p < 0.05, **p < 0.01, ***p < 0.001, ns = not significant.

We had previously shown that VCAM-targeted liposomes had improved brain delivery compared to non-specific controls in the ICH context, even at times of high vascular leakage^27^. However, we have also previously shown that intravenous LNPs can cause significant *in vivo* immune responses that can affect delivery and cargo expression^28^. Thus, our prior studies of LNP delivery in stroke mice relied on commercially sourced LNPs that were less amenable to modifications for our applications due to the proprietary nature of their composition. To this end, we re-engineered our VCAM-targeted LNPs using the ionizable lipid, 4A3-SC8 (Fig. 1A). These LNPs had similar size and conjugation efficiencies to our previously published liposomes and commercially acquired proprietary LNPs (Supplementary Fig. 1). We then tested whether the injured mice tolerated our new formulation, which led to similar observations as we had in the VCAM-targeted liposome counterpart. Mice were subjected to intrastriatal collagenase injection (Fig. 1B). Radiolabeled anti-VCAM-modified LNPs (as well as IgG-LNP controls) were injected at 4 hours post-injury and allowed to circulate for 30 minutes prior to harvesting. Using radiotracing, we observed that brain delivery of VCAM-targeted LNPs is ~ 10x higher than non-specific control (Fig. 1C) and is significantly higher in the injured hemisphere. Biodistribution to other organs is shown in Supplementary Fig. 2A, B; spleen biodistribution of VCAM-LNPs was significantly higher than control, as we have reported with other VCAM-targeted nanoparticles^27^. When normalized to blood, the localization ratio of VCAM-LNPs was 6x higher in the ipsilateral hemisphere (Supplementary Fig. 2C).

To test cargo expression, we generated LNPs containing luciferase mRNA and compared brain luminescence via VCAM-LNP delivery of the mRNA vs IgG control. We injected these LNPs at 2 hours post injury and allowed them to circulate for 4 hours, as prior studies have shown this is the time of highest expression of LNP-delivered mRNA^29,30^. We found that brain luminescence was ~ 10x higher in both hemispheres after ICH in the VCAM-LNP treated group compared to IgG control (Fig. 1D). In a separate experiment, we injected VCAM LNPs containing luciferase mRNA at 2 hours post-ICH and allowed them to circulate for 4 and 22 hours. The results show that at longer circulation times, brain luminescence was even higher after a single dose of VCAM-LNPs in the ipsilateral (injured) hemisphere (Fig. 1E). Taken together, these data confirm that our novel VCAM-targeted LNP formulation targets the ICH-affected brain and leads to higher expression of a candidate mRNA in the ICH-injured brains compared to non-specific control.

### Intrastriatal injection of collagenase forms the basis of a high-throughput screening paradigm for acute anti-inflammatory therapeutics.

**Figure 2:**
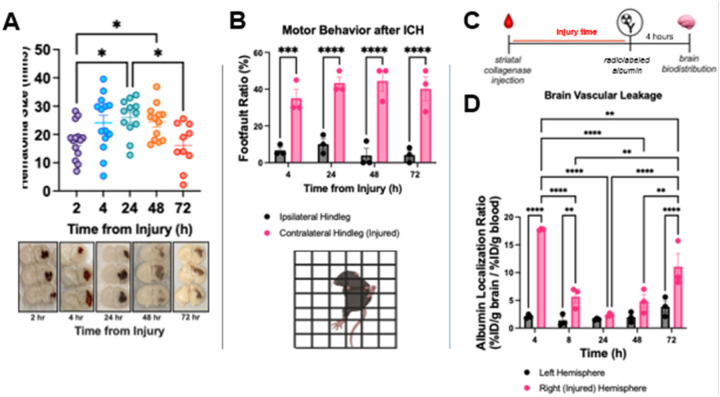
Establishment of high-throughput model for screening of promising therapeutics in ICH. (A) Quantification of hematoma volume (*top panel*) over time showed significant size increase between at 24 and 48hrs post-injury when compared to hematoma at 2 hrs post-injury, as visualized in gross pathology specimens (*bottom panel*) fixed in formalin and sliced into 1mm coronal sections. By 72 hours, hematoma is significantly decreasing in size. Data was analyzed via one-way ANOVA with post-hoc Tukey’s multiple comparisons test. (B)*Top panel*: Assessment of motor function as quantified by foot fault ratio demonstrated persistent motor deficits in contralateral limb (corresponding to injured hemisphere). *Bottom panel*: Graphical depiction of gridwalk test used to assess motor function. (C) Vascular leakage was measured by injecting radiolabeled albumin at various timepoints after ICH induction and allowing it to circulate for 4 hours prior to organ harvesting and gamma counting. (D) Analysis showed peak albumin leakage in right hemisphere (ipsilateral to the injury) at 4h post-injury, and later time points revealed bimodal distribution in albumin leakage with second peak readily apparent at 72h. All data represent mean ± SEM, *p < 0.05, **p < 0.01, ***p < 0.001, ns = not significant.

Intrastriatal injection of collagenase is one of the gold-standard methods of experimental ICH in murine models. Contrary to other models of cerebrovascular injury, this surgical approach is relatively uncomplicated and the collagenase dose can be titrated to a predictable phenotype with high survival, an important step in establishing a disease model for therapeutic screening. We previously showed that in our hands, this model leads to an initial expansion in hematoma size over 24 hours^25^ (similar to the timeline of hematoma expansion in humans^7,31,32^), followed by stable hematoma size up to 48 hours. Here, we expand these observations to show that hematoma size starts to decrease by 72 hours (Fig. 2A). Parallel to the hematoma and as expected, the mice develop a phenotype of hemiplegia on the contralateral side to the injury, and this deficit also remains stable over this timeframe (Fig. 2B). We had previously observed that in the collagenase model, radiolabeled albumin leaks into the brain over the first hours after ICH but resolves at 24 hours. We have now examined vascular leakage up to 72 hours after ICH via radiotracing of albumin into the brain (Fig. 2C). We observed that at this time point, there is a second wave of albumin leakage in the injured hemisphere at this time point (Fig. 2D), which likely represents post-ICH inflammation that could be a target for therapy. These three readouts (hematoma size, motor behavior, and vascular leakage) formed the basis of our high-throughput therapeutic screening strategy for promising LNP therapies in experimental ICH. Thus, we sought to test the platform by examining whether VCAM-LNP-mediated delivery of mRNA encoding an anti-inflammatory cytokine, IL-10, was sufficient to decrease injury size, decrease vascular leakage and improve motor behavior after ICH.

### VCAM-IL10 LNPs lead to reduced hematoma size and improved behavior.

**Figure 3:**
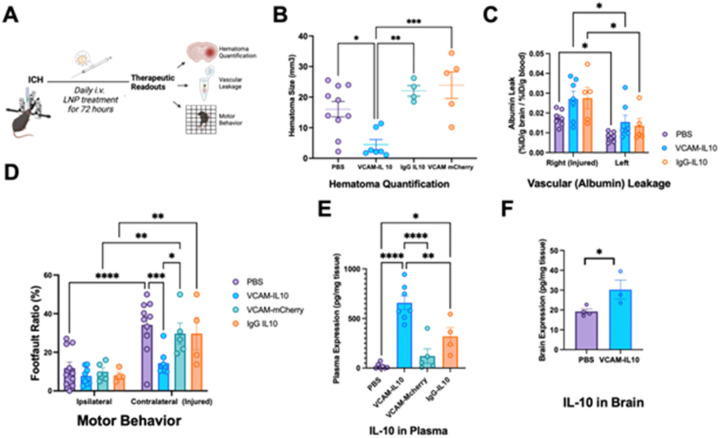
VCAM-targeted LNPs containing IL-10 mRNA are therapeutic in experimental ICH. (A) Schematic outlining the treatment regimen and therapeutic readouts. (B) Hematoma volume at 72 hours post-treatment shows that the VCAM-IL10 group experienced a notable reduction compared to all other groups. Data was analyzed via one-way ANOVA with post-hoc Tukey’s multiple comparisons test. (C) The albumin leak, calculated as the percentage of injected dose of radioactive albumin in the brain relative to the blood, revealed significant differences between ipsilateral and contralateral sides, suggesting significantly increased vascular leakage in the injured hemisphere, but no differences in leakage across treatment groups. (D) The foot fault ratio, representing motor function (number of missteps per total steps in 3 minutes), showed improvement in the VCAM-IL10 group compared to other groups. (E) IL-10 expression in plasma was higher in the VCAM-IL10 group relative to controls. In this panel, data was analyzed via one-way ANOVA with post-hoc Tukey’s multiple comparisons test. (F) IL-10 expression in the brain was significantly elevated in the VCAM-IL10 group compared to PBS-treated controls. Data analyzed via unpaired t-test. For all experiments, data represent mean ± SEM, **p < 0.01, ***p < 0.001, ****p < 0.0001, ns = not significant.

We tested whether VCAM-targeted LNPs containing IL-10 mRNA in our high-throughput ICH screening strategy. Based on data in the previous section, we focused on outcomes at 72 hours after injury. Considering the increased vascular leakage at 72 hours, we first chose that as the primary endpoint. Mice underwent ICH surgery and were injected with LNPs at 0.5h, 24hr, 48hr, and 68hrs after collagenase injection. Behavior was recorded prior to the last dose and scored blindly. Mice were subsequently sacrificed and their brains collected for measurement of vascular leakage via albumin radiotracing as well as blinded analysis of hematoma size (Fig. 3A). Control groups were treated with VCAM-targeted LNPs containing mCherry mRNA or untargeted LNPs (conjugated to nonspecific IgG) containing IL-10 mRNA. We observed that mice treated with VCAM-IL10 LNPs had significantly smaller hematoma sizes (Fig. 3B) and significantly improved motor behavior when compared to all other controls (Fig. 3D). There was no reduction in the amount of albumin leakage in either hemisphere in mice treated with VCAM-IL10 LNPs when compared to controls (Fig. 3C). At 72 hours, we also measured IL-10 expression in plasma (Fig. 3E) and in brain tissue (Fig. 3F), and found significantly higher levels in VCAM-IL10-LNP groups when compared to controls. There was no significant change to weight or kidney function among LNP treated groups. There was an increase in liver enzymes in the VCAM-mCherry-LNP control group (Supplementary Fig. 3).

### In the brain, VCAM-LNPs primarily target recruited leukocytes after ICH.

**Figure 4:**
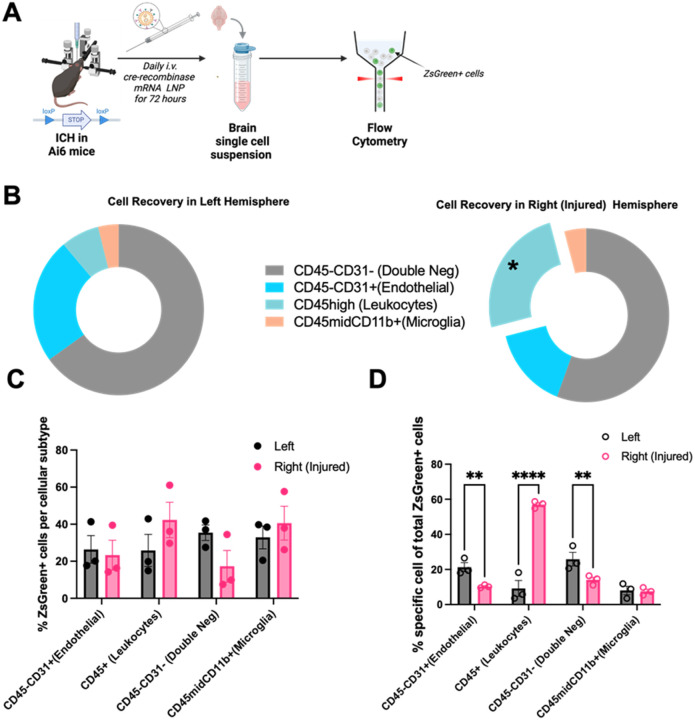
VCAM-LNPs in the ICH-injured hemisphere are primarily taken up by infiltrating leukocytes. (A) Ai6 mice have a loxP-flanked STOP sequence preventing expression of an enhanced green fluorescent protein variant, ZsGreen1+, which is expressed after Cre-mediated recombination. These mice were subjected to ICH surgery and subsequently treated with daily LNP (containing cre-recombinase mRNA) dosing for 72 hours, followed by brain homogenization for single cell suspension and analysis via flow cytometry. Thus, cells that take up LNPs and express cre-recombinase will also be ZsGreen+. (B) In the *left (non injured*) hemisphere, 54.7% of recovered cells were CD31−CD45− (double negative) cells, 19.93% were CD31+CD45− (endothelial) cells, 6.13% were CD45+ (leukocytes) cells, and 3.26% were CD45midCD11b+ (microglia) cells. In the *right* (injured) hemisphere, 50.77% of recovered cells were CD31−CD45− (double negative) cells, 14.03% were CD31+CD45− (endothelial) cells, 22.4% were CD45+ (leukocytes) cells (significantly higher than in the *left*hemisphere, *p=0.0272), and 3.89% were CD45midCD11b+ (microglia) cells. For panel B, data was analyzed via two-way ANOVA with post-hoc Sidak’s multiple comparisons test. (C) After gating for cellular populations, gating for ZsGreen+ signal was undertaken. Results represent %ZsGreen positive cells within specific cellular populations. No statistical significance in ZsGreen+ abundance within groups was seen among cellular groups or between hemispheres. (D) After identifying ZsGreen+ cells, gating for cellular populations was undertaken. Results show % of each specific cellular population from total ZsGreen+ cells. Leukocytes represent the majority of ZsGreen+ cells. For all experiments, data represent mean ± SEM, **p < 0.01, ***p < 0.001, ****p < 0.0001, ns = not significant.

To investigate which cellular populations were targeted within the ICH brain after VCAM-LNP treatment with our novel formulation, we obtained Ai6 mice which contain a loxP-flanked enhanced green fluorescent protein variant (ZsGreen1) that is expressed after Cre-mediated recombination. We generated VCAM-LNPs containing cre-recombinase mRNA and treated mice with daily doses for 3 days, as in our therapeutic paradigm. At the end of this time period, we harvested brains for immunohistochemistry and observed that most of the signal was confined to blood vessels (Supplementary Fig. 4). We then identified targeted cells via flow cytometry (Fig. 4A; gating strategy summarized in Supplementary Fig. 5). Among recovered cells (Fig. 4B), leukocytes were significantly more abundant in the right (injured) hemisphere when compared to the left. Among recovered cells, microglia (CD45midCd11b+) and leukocytes (CD45+) in the injured hemisphere were ZsGreen + at the highest rates (Fig. 4C) though differences in this metric were not statistically significant among evaluated cell types. In the injured hemisphere, double negative cells (CD31−CD45−) were ZsGreen + at a < 20% rate and were not characterized further. When first identifying Zgreen + cells, leukocytes represented the majority of cells expressing LNP cargo (Fig. 4D). For all animals, individual histograms for ZsGreen expression after gating for individual cell type are included in Supplementary Fig. 6.

### Macrophages are the most targeted leukocytes in the ICH brain after VCAM-LNP treatment.

Next, we examined individual leukocyte subpopulations. We primarily focused on characterizing cells that are known to be part of the initial innate immune response after ICH, and only broadly characterized lymphocytes for this experiment. Out of recovered leukocytes, macrophages (CD45 + CD64 + Ly6G−) represented the majority of recovered cells in the right (injured) hemisphere, and they were significantly more abundant than in the left hemisphere. We noted that CD64− neutrophils (non-activated neutrophils) expressed significantly more ZsGreen in the right (injured) hemisphere. However, when gating first by all ZsGreen + cells, the cell types that are thought to be key to the innate immune response after ICH (macrophages, microglia, and neutrophils) all express significantly more ZsGreen + in the ipsilateral hemisphere, suggesting they are the main drivers of expression induced by cargo mRNA from our novel VCAM-targeted LNPs.

## Discussion

ICH is a devastating form of stroke that has no disease-modifying therapeutic. In our prior work, we observed that targeted delivery to endothelium and leukocytes (using a targeting moiety against a commonly expressed adhesion molecule in these cells, VCAM) led to higher brain accumulation of a putative therapeutic protein. In this new manuscript, we sought to employ this strategy with a different kind of nanoparticle, LNPs, as these have great therapeutic potential to modulate protein expression via their nucleic acid cargos. Specifically, we wanted to see whether mRNA encoding the anti-inflammatory cytokine IL-10 (which has ample pre-clinical evidence supporting a potential therapeutic role in ICH) could be delivered via this strategy and confer better targeting and therapeutic effect than controls across different clinically-relevant outcomes.

To test this, we employed a novel LNP formulation developed by our lab, which uses an ionizable lipid, 4A3-SC8. Prior work from our lab had shown that this formulation reduces LNP-related side effects and increased cargo expression when compared to standard formulations^26^, but the formulation had never been employed in a brain injury model such as ICH. We show that, even in mice already ill with ICH, these LNPs can deliver and express mRNA cargo in the brain at significantly higher levels than a non-specific control. Further, the mice tolerated the treatment without significant changes to weight, liver or kidney function. Thus, given the arsenal of nucleic acid cargo that LNPs can carry to a target, these data set the stage for our subsequent therapeutic experiments.

Despite having a promising delivery strategy for ICH, it was important to consider what therapeutic outcomes can be measured that could truly screen for a promising therapeutic candidate. To this end, we designed a multimodal strategy to test both behavioral and tissue outcomes in our screening model. We considered three outcome measures in our design: hematoma size, motor behavioral outcome, and vascular leakage. We observed that our model had robust phenotypes in blinded experiments for all 3 of these variables and thus, would be sensitive enough to test for therapeutic effect. We initially hypothesized that delivery of anti-inflammatory cargo such as IL-10 would lead to a decrease in the albumin leakage we see after 72 hours in our model (and thus, in behavioral outcomes), as measured by radioactive albumin tracing into the brain, and chose 72 hours as the endpoint for our pilot therapeutic trial.

We delivered IL-10 mRNA via VCAM-targeted LNPs immediately after surgery and then daily until the 72 hour time point, for a total of 4 doses per animal. As controls, we also delivered IL-10 mRNA via LNPs conjugated to a non-specific IgG control, as well as VCAM-LNPs containing mCherry mRNA (as an untagged fluorescent protein that should have little or no role in modulating the immune response after ICH) and PBS treated controls. We observed that only that VCAM-IL10-LNP group had a therapeutic effect– mice in this group experienced a significant improvement in their hematoma size, and by consequence, in their motor behavior. Hematoma expansion is strongly correlated with worse outcomes after ICH^7,32–34^ and thus, many have sought to develop ICH therapies that target hematoma, such as platelet transfusions or promoting coagulation^35 36^, but these strategies have failed. Our data suggest that hematoma expansion is perhaps not just due to vessel rupture but that inflammation plays a role in this process. This idea is supported by other observations in literature showing that hemorrhagic transformation after ischemic stroke is modulated by infiltrating macrophages^37^ and that a high neutrophil-to-leukocyte ratio in ICH is also tied to hematoma expansion^38^. While inflammation has long been hypothesized as a targetable injury mechanism in subacute ICH, our data suggest that inflammation could also be a targetable mechanism in acute/hyperacute therapy.

Interestingly, we did not see a reduction in the magnitude of albumin vascular extravasation at 72 hours in the injured hemisphere in any of the treatment groups. This could be due to insufficient brain endothelial targeting by our targeting moiety in this disease model or insufficient dosing of IL-10, such that BBB restoration is not achieved within our experimental paradigm. A recent publication showed that increased IL-10 did lead to reduction in perihematomal edema^39^, but this was quantified via brain water content rather than direct extravasation. This technique reflects cumulative edema over time (including from other water sources such as CSF. Future experiments could test for albumin leakage in our experimental paradigm with other LNP doses or later timepoints, and also test for leakage of different-sized molecules beyond albumin. Despite persistent vascular leakage after treatments, our therapeutic VCAM-IL10-LNPs still led to improved motor behavior, confirming that reducing hematoma size is ultimately sufficient to affect changes to acute motor outcome after ICH, even without changes to vascular leakage.

We sought to confirm whether the cargo IL-10 mRNA was in fact expressed in treated animals. Indeed, mice treated with VCAM-IL10-LNPs had significantly higher plasma levels of IL10 when compared to both PBS treated and even IgG-IL10-LNP treated groups (Fig. 3E). We also saw a significant increase in IL-10 expression in the injured hemisphere of mice treated with VCAM-IL10 LNPs when compared to PBS control, but this effect was less robust than the peripheral elevations of IL-10 seen with our therapy. This observation underscores the role of the peripheral immune response in ICH outcomes, and raises hope that targeting of key cellular players within the peripheral immune system (a more feasible drug delivery goal than parenchymal targeting) could prove beneficial for ICH.

Our prior data in liposomes suggested that VCAM targeted LNPs would preferentially accumulate in the brain’s endothelium and infiltrated leukocytes. In our experimental paradigm, we observe that expression of LNP-encoded protein is highest among neutrophils and macrophages, followed by microglia and endothelial cells. It is possible that this is due to the specific experimental design using Ai6 mice, where one copy of cre-recombinase might be enough to modify a cell’s genome to express ZsGreen. Thus, repeated dosing is more likely to affect peripheral leukocytes, cells that are more rapidly dividing, compared to endothelial cells. Moreover, neutrophils and macrophages might also be phagocytosing ZsGreen that is released from neighboring cells, rather than directly producing ZsGreen due to targeting by VCAM-LNPs. Regardless, macrophages and neutrophils are key players in the post-ICH inflammatory response, and when considering that IL-10 is a secreted protein, the distribution of ZsGreen in this experiment likely represents the cells that are producing and being affected by IL-10, thus leading to the therapeutic response we see with our LNPs.

This work follows recently published data from our group that also showed therapeutic efficacy of IL-10 mRNA in an experimental model of ischemic stroke using a different formulation of commercially available LNPs targeted to VCAM^27^. While ischemic and hemorrhagic stroke are separate diseases, there are common elements to the immune response in both that suggest a common therapeutic could work to modulate neuro-inflammation in either form of stroke. An advantage of a therapeutic such as the one we propose here is the potential for very early administration in the clinical context, as it may not be necessary to discriminate between type of stroke prior to intervention.

This manuscript is among the first in the field to describe a therapeutic response in an experimental ICH model using intravenously delivered mRNA LNPs. The data presented here lay the groundwork for future therapeutic experiments using a wide array of mRNA (or even combinations of mRNA) to modulate the immune response after ICH, particularly considering the accessibility of the blood brain barrier (brain endothelium and recruited peripheral leukocytes) as a target in this model.

## Conclusions

We show that VCAM LNPs lead to significantly higher brain expression of cargo mRNA in experimental ICH, when compared to non-specific control. We leverage this targeting strategy to deliver IL-10 mRNA after ICH and show this has therapeutic effect in decreasing hematoma size and improving motor behavior. Our nanoparticles preferentially express in infiltrated leukocytes in the ICH brain, of which macrophages form the biggest component. These data are among the first to report therapeutic efficacy of mRNA LNPs in experimental ICH, a disease in which the human counterpart has limited therapies and high mortality.

## Figures and Tables

**Figure 5:**
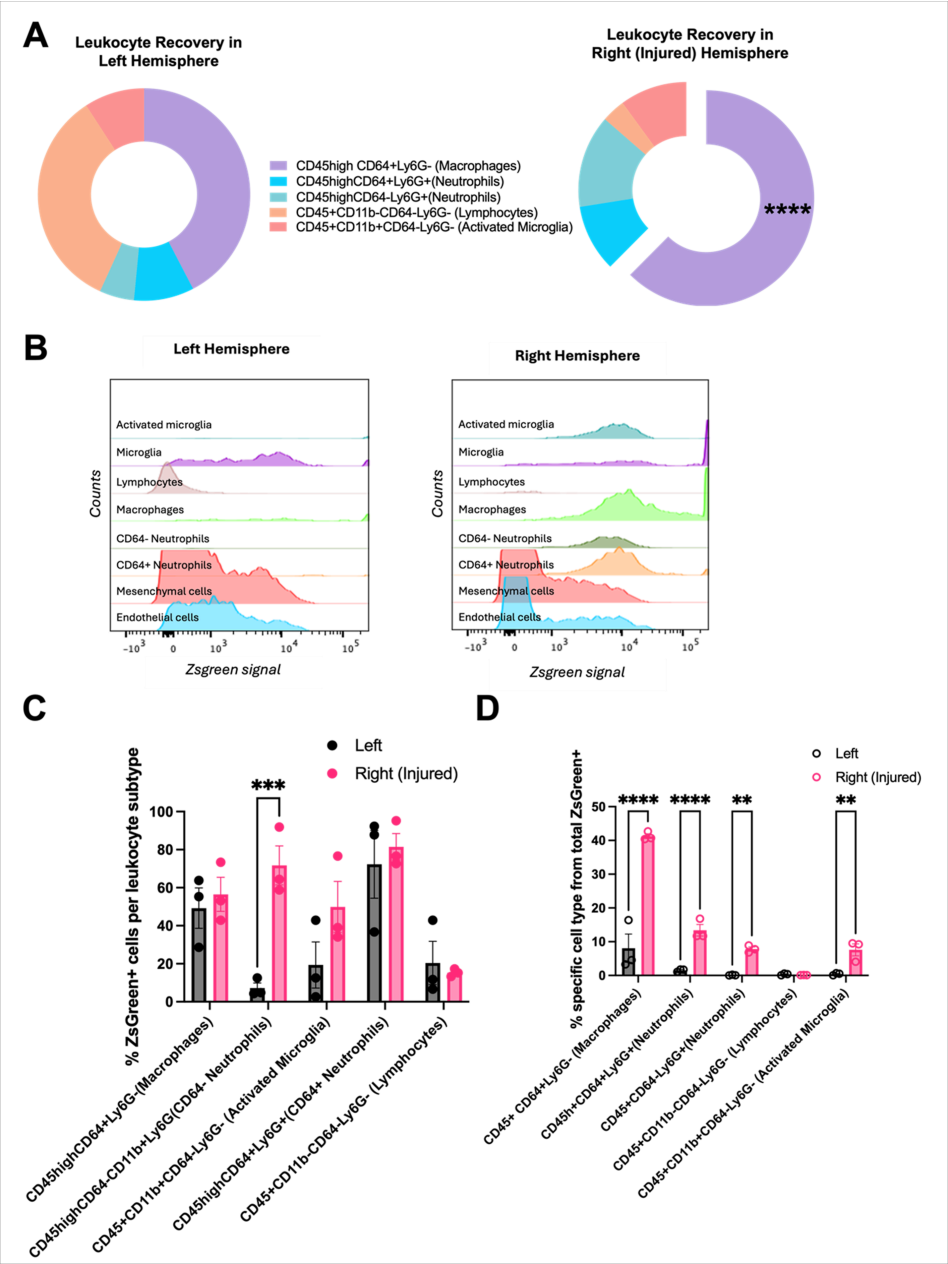
Macrophages are the dominant leukcotye taking up VCAM-LNPs in the ICH-injured brain. Following gating for CD45, leukocytic populations were further characterized via flow cytometry as in Figure 4. (A) In the *left* hemisphere, 2.83% of all recovered cells (35.4% of CD45high cells) were macrophages, 2.27% (32.6%) were lymphocytes, 0.61 (9.2%) were activated microglia, 0.58% (14.9%) were CD64+ neutrophils, and 0.35% (6.3%) were CD64− neutrophils. In the *right (injured)* hemisphere, 15.43% of all recovered cells (47.6% of CD45high cells) were macrophages (significantly higher than the left hemisphere, ****p<0.0001), 0.88% (3.5%) were lymphocytes, 2.5 (12.0%) were activated microglia, 4.8% (21.9%) were CD64+ neutrophils, and 3.4% (14.8%) were CD64− neutrophils. For panel A, data was analyzed via two-way ANOVA with post-hoc Sidak’s multiple comparisons test. (B) An example of the histogram for a single mouse is shown. After gating for individual cell populations in this specimen, ZsGreen signal for each cell type is shown on the x-axis. Histograms for all animals in the experiment are shown in Supplementary Figure 6. (C) After gating for leukocyte subpopulations, gating for ZsGreen+ signal was undertaken. Results represent %ZsGreen positive cells within specific cellular populations. Significantly higher levels of ZsGreen expression were seen among CD64− neutrophils. (D) After identifying ZsGreen+ cells, gating for cell subtypes was undertaken. Results show % of each specific cellular population among total ZsGreen+ cells. Macrophages represent the majority of ZsGreen+ leukocytes, in keeping with them being the leukocytic group with highest cell recovery. For all experiments, data represent mean ± SEM, **p < 0.01, ***p < 0.001, ****p < 0.0001, ns = not significant.

**Figure 6:**
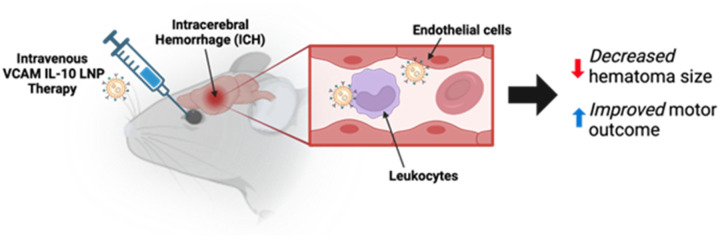
Graphical Abstract. Intravenous therapy with VCAM-LNPs containing IL-10 mRNA leads to targeting of leukocytes associated with the innate immune response after ICH, which results in decreased hematoma size and improved motor outcomes after ICH.

## Data Availability

The relevant datasets supporting the conclusions of this article are included within the article (and its additional files). Any additional data is available from the corresponding author upon reasonable request.

## Abbreviations

ICH: Intracerebral hemorrhage
LNP: Lipid nanoparticle
VCAM: Vascular cellular adhesion molecule
BBB: Blood brain barrier
DBCO: Dibenzocyclooctyne
PDI: Polydispersity index
DLS: Dynamic light scattering
NTA: Nanoparticle tracking analysis
